# Causes of severe infections in patients with systemic sclerosis and associated factors

**DOI:** 10.55730/1300-0144.5535

**Published:** 2022-10-10

**Authors:** Müçteba Enes YAYLA, Emine USLU YURTERİ, Murat TORGUTALP, Didem ŞAHİN, Serdar SEZER, Ayşe Bahar KELEŞOĞLU DİNÇER, Emine Gözde AYDEMİR GÜLÖKSÜZ, Mehmet Levent YÜKSEL, Recep YILMAZ, Aşkın ATEŞ, Tahsin Murat TURGAY, Gülay KINIKLI

**Affiliations:** 1 Department of Internal Medicine, Division of Rheumatology, Ankara Training and Research Hospital, Ankara, Turkey; 2 Department of Internal Medicine, Division of Rheumatology, Faculty of Medicine, Ankara University, Ankara, Turkey

**Keywords:** Severe infection, systemic sclerosis, digital ulcer, cardiac involvement

## Abstract

**Background/aim:**

Systemic sclerosis (SSc) is a chronic systemic disease characterized by vascular damage, autoimmunity, and fibrosis in the skin and internal organs. In this study, we tried to determine the causes of severe infection in patients with SSc and to reveal the factors associated with severe infection.

**Materials and methods:**

We retrospectively examined 214 SSc patients between January 2010 and August 2020. Forty-seven patients with at least one severe infection and 167 patients without severe infection were compared.

**Results:**

A total of 76 episodes of severe infections were detected in 47 (22%) patients. Common infections included pneumonia, infected digital ulcer, urinary tract infections, and osteomyelitis. Female patients had a higher frequency in the group without severe infection (91.6% vs. 80.9%, p = 0.035). Patients with severe infections had a higher frequency of digital ulcers (p < 0.001), cardiac (p = 0.002), and GIS involvement (p < 0.001). In multivariable analysis, digital ulcer presence (OR: 2.849 [1.356–5.898] (p = 0.006) and cardiac involvement (OR: 2.801 [1.248–6.285]) were associated with severe infection. Of the patients with severe infections, 34% had recurrent severe infections. There was no difference in demographic and clinical characteristics between patients with recurrent and nonrecurrent severe infections.

**Conclusion:**

The presence of digital ulcer and cardiac involvement seem to be associated with a severe infection in patients with systemic sclerosis. In patients with cardiac involvement and digital ulcers, more careful attention may be required for the development of severe infections.

## 1. Introduction

Systemic sclerosis (SSc) is a chronic systemic disease characterized by vascular damage, autoimmunity, and fibrosis in the skin and internal organs. In SSc patients, a wide spectrum of clinical manifestations could be seen, from limited skin involvement and Raynaud’s phenomenon to life-threatening organ involvement [ 1 ]. Infections are an important cause of hospitalizations, morbidity, and mortality in patients with SSc [ 2–4] . In SSc, infections can be seen with an incidence rate of 20.3 per 100 person years, of which more than half are serious infections [ 4 ]. In addition, the involvement of vital internal organs and the use of strong immunosuppressive therapies increase the frequency of infections [ 5 ]. Female gender, Raynaud’s phenomenon, and especially esophageal dysmotility can be considered among the important risk factors for the prediction of infections [ 4 ].

In this study, we tried to determine the causes of severe infection in patients with SSc and to reveal the factors associated with severe infection.

## 2. Materials and methods

### 2.1. Patient selection

We retrospectively examined 214 patients with SSc over the age of 18 years who applied to Department of Rheumatology between January 2010 and August 2020 and who met the 2013 ACR/EULAR systemic sclerosis classification criteria [ 6 ]. Severe infection was defined as a condition that required intravenous antibiotic usage or hospitalization or resulted in death, according to previous studies [ 7–10] . The diagnosis of infection was made by internal medicine specialists or infectious disease specialists, or rheumatologists. The type of infection was determined after anamnesis, physical examination, and laboratory tests. In this study, 47 patients with at least one severe infection and 167 patients without a history of severe infection were compared.

### 2.2. Definition of demographic and clinical features

The age of disease onset was accepted as the time of onset of the first non–Raynaud’s disease manifestation. The duration of the disease was accepted as the time between the date of the first severe infection and the onset of the disease in the severe infection group, whereas the time between the date of the last visit and the onset of the disease in the noninfection group. The duration of the follow-up was defined as the time between the dates of the last and first visits.

The patients were classified into diffuse cutaneous SSc (dcSSc), limited cutaneous SSc (lcSSc) based on the extent of skin involvement [ 11 ] and sine-scleroderma (with no skin thickening on physical examination). Diagnosis of interstitial lung disease (ILD) was established by the presence of at least one of the classical pulmonary manifestations, such as subpleural opacity, interstitial reticular pattern, and honeycomb appearance on chest computed tomography (Chest CT) [ 12 ]. Pulmonary arterial hypertension (PAH) in right heart catheterization was defined as a mean pulmonary pressure of 25 mmHg and above and a pulmonary capillary wedge pressure of 15 mmHg or less [ 13 ]. The presence of cardiac involvement was determined by the detection of arrhythmia, pericardial effusion, pericardial thickening, or valvular heart disease, which could not be explained by any other etiological reasons, on electrocardiography or echocardiography [ 14 ]. The presence of gastrointestinal system (GIS) involvement was determined by signs indicating impaired motility on manometric tests or endoscopic interventions, or by enlarged esophagus in chest CT. The presence of multi-organ/system involvement was defined as patients in which two or more systems or organs were affected. Immunosuppressive treatments and their doses (treatments and doses until the onset of infection in patients with severe infection) used during the entire follow-up period were recorded. In the severe infection group, the types of infections and the number of infection episodes were recorded.

The demographic, clinical and laboratory characteristics of SSc patients with and without severe infection were compared. The study was reviewed and approved by Clinical Research Ethics Committee (No: İ8-541-20 Date: 09.28.2020) and conducted in accordance with the ethical standards of the Helsinki Declaration.

### 2.3. Statistical analysis

The data were evaluated using the software IBM SPSS version 21 (Chicago, USA). Quantitative data were expressed as median and interquartile range (IQR). Fisher’s exact test or Chi-square test, whichever is appropriate, was used for the comparison of categorical data and Mann Whitney U test was used to compare continuous data between groups with and without severe infection. Multivariable analysis was performed by logistic regression analysis, which modeled independent predictors to predict severe infections, by using possible factors identified in univariate analyses. (Variables with a p-value < 0.1 on univariate analysis were subsequently entered into the final multivariable model). Hosmer-Lemeshow test was used to assess goodness-of-fit. In the analyses, p value under 0.05 was accepted as statistically significant.

## 3. Results

A total of 76 episodes of severe infections were detected in 47 (22%) patients. Common infections included pneumonia, infected digital ulcer, urinary tract infections, and osteomyelitis. The cumulative severe infection types are presented in Table 1.

Patients with and without severe infection were similar in terms of age (p = 0.744), duration of disease (p = 0.138), and follow-up time (p = 0.118). Female patients had a higher frequency in the group without severe infection (91.6% vs. 80.9%, p = 0.035). Patients with severe infections had a higher frequency of digital ulcers (p < 0.001), cardiac (p = 0.002), GIS involvement (p < 0.001) and multiple organ/system involvement (p < 0.001). The patients with dcSSc were more common in the group with severe infection, but there was no statistical significance (p = 0.051) (Table 2). There was no difference between the groups with and without severe infection in the frequency and doses of immunosuppressive therapy, except for cyclophosphamide. Patients with severe infections had a higher rate of cyclophosphamide use (p = 0.018), but with the absence of statistically significant difference in the cumulative doses between two groups (p = 0.195) (Table 2).

Univariate and multivariable logistic regression analysis are presented in Table 3. In multivariable analysis, digital ulcer presence (OR: 2.849 [1.356–5.898] (p = 0.006) and cardiac involvement (OR: 2.801 [1.248–6.285]) were associated with severe infection (Table 3).

Of the patients with severe infections, 34% (16/47) had recurrent severe infections. There was no difference in demographic, clinical, and therapeutic characteristics between patients with recurrent and nonrecurrent severe infections (Table 4).

Considering the frequency of severe infections based on the involvement of major organs/systems, patients with digital ulcer and cardiac involvement had the highest frequency of skin and soft tissue infections, and patients with interstitial lung disease, pulmonary arterial hypertension, and GIS involvement had the highest frequency of pneumonia (Table 5).

A total of 11 patients had died during the follow-up period. There was a higher mortality rate in the severe infection group (9 (%19.1) vs. 2 (%1.2), p < 0.001). In addition, the causes of death were respiratory failure secondary to ILD in 3 patients, cardiovascular event in 2 patients, malignancy (B-cell lymphoma and gastric carcinoma) in 2 patients, sepsis secondary to osteomyelitis in 1 patient, and PAH-related respiratory failure in 1 patient in severe infection group. In the group without severe infection, the causes of deaths were PAH-related respiratory failure in 2 patients.

## 4. Discussion

It is known that there is an increased frequency of infections in connective tissue diseases due to the disease itself and immunosuppressive treatments [ 15 ]. However, there are a limited number of studies of this subject on patients with systemic sclerosis, which have shown that infections can be frequently observed [3, 4, 15 ]. In this study, we tried to show the causes of severe infection in patients with SSc and possible related clinic factors.

In systemic sclerosis, infections can have major effects on the frequency and duration of hospitalization and mortality rates [16, 17]. Therefore, determining the factors that predict the development of infections may be important for clinicians to follow up their patients. Previous studies have indicated that Raynaud’s phenomenon, digital ulcer, ILD, and GIS involvement are among the predictors of infections in SSc patients [ 15 ]. Our study showed a relationship between severe infections and male gender, digital ulcer, GIS and cardiac involvement and use of cyclophosphamide in univariate analyzes, and digital ulcer presence and cardiac involvement in multivariable analyses (Table 3).

There is a limited number of studies investigating the causes of severe infections in SSc. A recent study reported that pneumonia had the highest frequency (45%) in SSc patients, followed by sepsis (32%), skin and soft tissue infections (19%), and urinary tract infections (3%) [ 3 ]. Another study conducted in Taiwan reported that skin and soft tissue infections were the most common causes, followed by GIS infections, urinary tract infections, and pneumonia [ 4 ]. In the present study, the most common infections included skin and soft tissue infections, particularly digital ulcer infection and osteomyelitis, pneumonia, and urinary tract infections, consistent with previous studies (Table 1). Our study also examined the effects of SSc involvement in organs/systems on types of infections (Table 5). Skin and soft tissue infections were more common in patients with digital ulcer and cardiac involvement, whereas pneumonia was higher in patients with ILD, PAH and GIS involvement. A higher frequency of infected digital ulcer and osteomyelitis can be expected in those with digital ulcers. Microvascular damage is known to occur in SSc [ 18 ]. In addition, the increase in the frequency of digital ulcers and osteomyelitis in patients with cardiac involvement suggests that circulatory deterioration due to both microvascular and macrovascular reasons may have set the ground for infections as a result of delayed tissue healing. Therefore, we may have found a relationship between severe infections and digital ulcer and cardiac involvement in multivariable analyses (Table 3). Similarly, it has been suggested that conditions associated with pulmonary fibrosis, such as ILD, may pose a risk for the development of infection associated with lung tissue damage. It is also known that the frequency of aspiration pneumonia increases in patients with SSc due to lower esophageal sphincter dysfunction [ 15 ]. Therefore, we expected an increased frequency of pneumonia in patients with ILD or GIS involvement. Moreover, our data showed an association between GIS involvement and the presence of severe infection in univariate analysis, in line with previous studies [4, 15, 19, 20 ]. However, this relationship did not reach statistical significance in multivariable analyses. In SSc, GIS involvement causes aspiration pneumonia as well as excessive bacterial proliferation, ileus and malabsorption due to hypomotility which is a result of intestinal fibrosis, thus setting the ground for infections [ 21, 22 ].

In our study, although male gender was found to be a risk factor for the development of severe infections in univariate analyses, no significant result was obtained in multivariable analyses. The higher frequency of digital ulcers in our male patients may have caused this result. As far as we know, there is only one study examining the effect of gender on the development of infections in SSc. Although that study reported a 2.3–fold increase in the risk of developing infections in women, it also reported the absence of any effect of sex on major infections [ 4 ]. We believe that more comprehensive studies are needed to evaluate the effect of gender on infections.

The use of immunosuppressants may be a risk factor for the development of infections in autoimmune diseases [ 15 ]. The use of glucocorticoids has been shown to be a risk factor for the development of severe infections, particularly in a dose dependent manner [ 15, 23, 24 ]. In SSc, glucocorticoids are generally used at lower doses due to the risk of triggering renal crises. In our center, the daily dose generally does not exceed the equivalent of 7.5 mg of prednisolone. The lack of increased risk of serious infections in glucocorticoid users can be attributed to this dose restriction. Furthermore, cyclophosphamide is an important therapeutic agent, especially in SSc patients with severe organ involvement. After the use of cyclophosphamide, the risk of severe infections has been found to be increased in other autoimmune diseases [ 15, 25 ]. We also found a higher frequency of cyclophosphamide use in patients with severe infections, but in multivariable analysis it could not be shown to be associated with severe infections. Moreover, we found a lack of a total dose-dependent effect of cyclophosphamide on infections. We also did not detect an opportunistic infection in our patients that may occur due to the immunosuppressive effect of cyclophosphamide.

This study has several limitations, such as having a single-center retrospective study design and absence of disease activity and severity assessments of the patients. We could not evaluate all the potential confounding factors for infection risk such as tobacco and alcohol use, vaccination practice, surgery, pet ownership, hospitalization due to noninfection reasons and comorbidities. Since our current study has a single-center retrospective study design, we may have been insufficient to obtain the data of patients who presented to another health center with severe infections. In addition, it has the strengths of being one of the few studies examining factors associated with severe infections in SSc patients.

In conclusion, the presence of digital ulcer and cardiac involvement seem to be associated with severe infection in patients with systemic sclerosis. In patients with cardiac involvement and digital ulcers, more careful attention may be required for the development of severe infections.

## Figures and Tables

**Table 1:** Causes of severe infections.

		Cumulative infections*
Skin and soft tissue infections		30 (39.5)
	Infected digital ulcer	16 (21)
	Osteomyelitis	12 (15.8)
	Infected pressure ulcers	2 (2.6)
Pneumonitis		22 (28.9)
Urinary tract infections		15 (19.7)
Gastrointestinal infections		4 (5.3)
Others		5 (6.6)
	Ear, nose, and throat infections	3 (3.9)
	Bacterial spondylodiscitis	1 (1.3)
	Renal tuberculosis	1 (1.3)
Total		76

* Number and percent

**Table 2:** Comparison of patients with and without severe infection in terms of demographic and clinical features.

	Severe infection group N = 47	No infections N = 167	p
Age, years*	52.1 [21.6]	53 [20.7]	0.744
Gender, female†	38 (80.9)	153 (91.6)	**0.035**
Age of disease onset, year*	42.2 [20.5]	45.6 [18.4]	0.128
Disease duration, year*	8,5 [13.75]	6.6 [9.7]	0.138
Follow-up time, year*	5.5 [5.5]	4.25 [5]	0.118
**Clinical features** †			
Raynaud phenomenon	45 (95.7)	158 (94.6)	1
Digital ulcer	29 (61.7)	49 (29.3)	**<0.001**
Interstitial lung disease	26 (55.3)	73 (43.7)	0.159
Pulmonary arterial hypertension	5 (10.6)	7 (4.2)	0.143
Cardiac involvement	18 (38.3)	29 (17.5)	**0.002**
Gastrointestinal involvement	23 (48.9)	38 (22.9)	**<0.001**
Arthritis/arthralgia	9 (19.1)	24 (14.5)	0.433
Multiple organ/system involvement	33 (70.2)	56 (33.5)	<0.001
**Classification** †			
dcSSc	17 (36.2)	37 (22.2)	0.051
lcSSc	30 (63.8)	125 (74.9)	0.135
Sine-scleroderma	0	5 (3)	0.588
**Immunosuppressive treatments**			
Glucocorticoids†			
Usage, ever	22 (46.8)	57 (34.1)	0.112
Usage, current	18 (38.3)	46 (27.5)	0.155
Total dose, mg*	4100 [4537.5]	6300 [10,975]	0.281
Azathioprine†			
Usage, ever	7 (14.9)	24 (14.4)	0.928
Usage, current	4 (8.5)	18 (10.8)	0.790
Total dose, g*	129 [588]	85.5 [108.4]	0.304
Mycophenolate mofetil†			
Usage, ever	5 (10.6)	17 (10.2)	1
Usage, current	5 (10.6)	15 (9)	0.777
Total dose, g*	630 [735]	1125 [1478.8]	0.275
Cyclophosphamide†			
Usage, ever	11 (23.4)	17 (10.2)	**0.018**
Usage, current	2 (4.3)	1 (0.6)	0.122
Total dose, g*	9 [6]	6 [4.45]	0.195
Rituximab†			
Usage, ever	0	7 (4.2)	0.352
Usage, current	0	4 (2.4)	0.578
Total dose, g*	-	2 [13]	-

dcSSc; diffuse cutaneous systemic sclerosis, g; gram, lcSSc; limited cutaneous systemic sclerosis, mg; milligram.

* Data are presented as median and IQR.

† Data are presented as numbers and percent.

**Table 3:** Associated factors for severe infection.

	Univariate analysis		Multivariable analysis*	
	OR (95% CI)	p	OR (95% CI)	p
Age, year	1.006 [0.983–1.029]	0.613	-	-
Gender, male	2.588 [1.042–6.428]	0.040	2.280 [0.791–6.574]	0.127
Disease duration, year	1.040 [0.999–1.082]	0.057	1.005 [0.958–1.005]	0.838
Digital ulcer presence	3.880 [1.974–7.627]	<0.001	2.849 [1.356–5.898]	**0.006**
Interstitial lung disease	1.594 [0.831–3.058]	0.160	**-**	**-**
Pulmonary arterial hypertension	2.704 [0.817–8.951]	0.103	**-**	**-**
Cardiac involvement	2.932 [1.439–5.974]	0.003	2.801 [1.248–6.285]	**0.013**
Gastrointestinal involvement	3.228 [1.640–6.352]	0.001	2.098 [0.967–4.550]	**0.061**
dcSSc	1.991 [0.991–4.002]	0.053	0.789 [0.331–1.897]	0.592
Glucocorticoid usage, ever	1.698 [0.881–3.273]	0.144	-	-
Cyclophosphamide usage, ever	2.696 [1.163–6.252]	0.021	2.134 [0.775–5.880]	0.143

CI; Confidence interval, dcSSc; diffuse cutaneous systemic sclerosis, OR; Odds ratio,

* For multivariable model; Hosmer and lemeshow test chi-square 2.468, p = 0.963.

**Table 4:** Comparison of patients with and without recurrent severe infection in terms of demographic, and clinical features.

	Recurrent severe infection group N = 16	Single severe infection group N = 31	P
Age, years*	59 [25.2]	49.75 [22.1]	0.119
Gender, female†	15 (93.8)	23 (74.2)	0.138
Age of disease onset, year*	44 [25.3]	36.5 [22.4]	0.307
Disease duration, year*	8.2 [12.3]	8.8 [15.7]	0.694
Follow-up time, year*	7 [8.1]	5 [4.7]	0.138
**Clinical features** †			
Raynaud phenomenon	15 (93.8)	30 (96.8)	1
Digital ulcer	11 (68.8)	18 (58.1)	0.475
Interstitial lung disease	10 (62.5)	16 (51.6)	0.477
Pulmonary arterial hypertension	1 (6.3)	4 (12.9)	0.648
Cardiac involvement	7 (43.8)	11 (35.5)	0.581
Gastrointestinal involvement	8 (50)	15 (48.4)	0.917
Arthritis/arthralgia	5 (31.3)	4 (12.9)	0.239
Multiple organ/system involvement	12 (75)	21 (67.7)	0.742
**Classification** †			
dcSSc	3 (18.8)	14 (45.2)	0.074
lcSSc	13 (81.3)	17 (54.8)	0.074
Sine-scleroderma	0	0	-
**Immunosuppressive treatments**			
Glucocorticoids†			
Usage, ever	8 (50)	14 (45.2)	0.753
Usage, current	6 (37.5)	12 (38.7)	0.936
Total dose, mg*	4100 [2250]	4081 [8487.5]	0.971
Azathioprine†			
Usage, ever	2 (12.5)	5 (16.1)	1
Usage, current	1 (6.3)	3 (9.7)	1
Total dose, g*	135 [−]	129 [711.5]	1
Mycophenolate mofetil†			
Usage, ever	0	5 (16.1)	0.150
Usage, current	0	5 (16.1)	0.150
Total dose, g*	-	630 [735]	-
Cyclophosphamide†			
Usage, ever	4 (25)	7 (22.6)	1
Usage, current	1 (6.3)	1 (3.2)	1
Total dose, g*	9 [51.75]	6.75 [6]	0.788

dcSSc; diffuse cutaneous systemic sclerosis, g; gram, lcSSc; limited cutaneous systemic sclerosis, mg; milligram.

* Data are presented as median and IQR.

† Data are presented as numbers and percent.

**Table 5:** Severe infection event frequencies according to organ involvement.

	Digital ulcer	Interstitial lung disease	Pulmonary arterial hypertension	Gastrointestinal involvement	Cardiac involvement	Multiple organ/system involvement
Skin and soft tissue infections*	27 (52.9)	9 (20.4)	1 (12.5)	14 (35)	14 (41.2)	23 (41.1)
Pneumonitis*	15 (29.4)	19 (43.2)	4 (50)	16 (40)	13 (38.2)	19 (33.9)
Urinary tract infections*	7 (13.7)	10 (22.7)	2 (25)	4 (10)	6 (17.6)	8 (14.3)
Gastrointestinal infections*	0	3 (6.8)	1 (12.5)	3 (7.5)	0	3 (5.4)
Others*	2 (3.9)	3 (6.8)	0	3 (7.5)	1 (2.9)	3 (5.4)
Total	51	44	8	40	34	56

* Event number and percent.
